# Optimizing microfluidic chip for rapid SARS-CoV-2 detection using Taguchi method and artificial neural network PSO

**DOI:** 10.1038/s41598-025-98304-5

**Published:** 2025-04-23

**Authors:** Sameh Kaziz, Fraj Echouchene, Mohamed Hichem Gazzah

**Affiliations:** 1 NANOMISENE Laboratory, LR16CRMN01, Centre for Research on Microelectronics and Nanotechnology (CRMN) of Sousse Technopole, Sousse, Tunisia; 2 Higher Institute of Applied Sciences and Technology of Sousse, University of Sousse, Ettafala City, Ibn Khaldoun, Sousse, 4003 Tunisia; 3 Laboratory of Electronics and Microelectronics LR99ES30, Faculty of Sciences, University of Monastir, Monastir, 5000 Tunisia; 4 Quantum and Statistical Physics Laboratory, Faculty of Sciences of Monastir, University of Monastir, Environment Boulevard, Monastir, 5019 Tunisia

**Keywords:** ANOVA, Biosensor, SARS-CoV-2, Particle swarm optimization, Taguchi method, Biophysics, Computational biology and bioinformatics

## Abstract

Microfluidic biosensors offer a promising solution for real-time analysis of coronaviruses with minimal sample volumes. This study optimizes a biochip for the rapid detection of SARS-CoV-2 using the Taguchi orthogonal table L_9_(3^4^), which comprises nine groups of experiments varying four key parameters: Reynolds number (Re), Damköhler number (Da), Schmidt number (Sc), and the dimensionless position of the reaction surface (X). Signal-to-noise (S/N) ratios and analysis of variance (ANOVA) are employed to determine optimal parameters and assess their impact on binding kinetics and response time of the detection device. These obtained optimal parameters correspond to Re = 4.10^-2^, Da = 1000, Sc = 10^5^, and X = 1. Additionally, results highlight Da as the most influential factor, accounting for 91%, while X has a minimal effect of 0.3%. Furthermore, an artificial neural network optimization technique, specifically particle swarm optimization (PSO), was utilized to predict biosensor performance. Derived from the Full L_81_(3^4^) design experiment, the PSO model demonstrates its effectiveness compared to the conventional multi-layer perception (MLP) model, thus underlining its potential in this innovative optimization context.

## Introduction

Early detection of COVID-19 is crucial for effective pandemic management, enabling rapid isolation, preventing disease spread, and facilitating early treatment. The primary method for detecting SARS-CoV-2 is real-time reverse transcription polymerase chain reaction (RT-PCR)^1,2^. However, RT-PCR diagnostics require expensive reagents, specialized equipment, and trained personnel^3^. While conventional RT-PCR is often time-consuming, recent advancements in microfluidic-based PCR have significantly improved speed, cost-effectiveness, and sensitivity^4^. These techniques offer rapid amplification, reduced reagent consumption, and lower detection limits, making them competitive with traditional methods^5^.

To further reduce detection time, various point-of-care (POC) biosensors have been developed^1,6–8^, targeting antigens, antibodies, or nucleic acids^7^. While antibody tests are suitable for late-stage infections, nucleic acid-based detection methods are preferred for early- stage diagnosis due to their superior sensitivity and specificity. However, nucleic acid testing involves more complex processes such as extraction, amplification, and detection^9^. Although RT-PCR remains clinically more sensitive and specific than POC biosensors, immunodiagnostic assays offer a reliable and cost-effective alternative by improving specific immunoassays for desired antigen proteins.

Immunoassays, based on antigen-antibody interactions, have garnered significant interest in fields such as medicine and environmental monitoring^10^. Traditional immunoassays include complex detection protocols and require skilled professionals. Moreover, diffusion-limited reaction kinetics and lengthy incubation steps limit their broad applications^11^. To address unmet medical needs like early and rapid disease diagnosis, immunoassays are increasingly being adapted to microfluidic formats^12^. This miniaturization technology enhances analysis performance by integrating multiple processes into a single chip, reducing analysis time and increasing sensitivity and reliability with minimal reagent use^13^. Microfluidic chip technology allows for the simultaneous detection of different samples, which is valuable in protein chips. However, its use is limited by the diffusion transport of antigens in laminar flow, where low antigen concentrations delay detection^14^. To improve the sensitivity of microfluidic chips, many numerical and experimental studies have been conducted. Various physical mechanisms, such as magnetic effects^15^, optical forces^16^, and electrokinetic effects^17–19^, have been applied to enhance flow agitation and biosensor binding reaction rates. Other studies^19,20^ have analyzed the effects of reaction surface and electrodes shapes on biosensor performance. Shahbazi et al.^21^ demonstrated that the reaction surface’s location relative to the channel inlet significantly impacts microfluidic biosensor efficiency.

In related fields, recent studies have explored novel techniques for rapid and sensitive detection of various analytes, underscoring the necessity for continuous development in this area^22–24^. Among these advancements, the use of nanomaterials in biosensing has shown great potential in various applications. Nanomaterial-based electrochemical biosensing has been effectively employed to detect fumonisins, demonstrating the importance of developing sensitive detection techniques^25^. Furthermore, the use of metal-organic frameworks (MOFs) for electrochemical-based sensing platforms has shown promising results in detecting glucose and hydrogen peroxide^26^. These advancements illustrate the broader applicability and importance of developing specialized assays for different analytes.

Other research has identified several factors influencing the kinetics of antigen-antibody binding reactions in microfluidic biosensors, including flow velocity, target antigen concentration, reaction surface position, microfluidic dimensions, and biosensor shape. Optimizing these factors is crucial for enhancing immunoassay performance. Taguchi’s experimental design is a well-known technique for process optimization, providing a systematic and efficient methodology^27–29^. Taguchi’s method aims to design quality into the product by optimizing control factors through simple tools like signal-to-noise ratio (S/N) and analysis of variance (ANOVA)^28^.

Additionally, integrating artificial intelligence (AI) and machine learning has become prominent in optimizing microfluidic biosensor performance. Machine learning algorithms address complex and nonlinear problems, aiding in rapid and accurate data analysis and optimization. Specifically, Particle Swarm Optimization (PSO) combined with Artificial Neural Networks (ANN-PSO) has been applied to enhance detection device performance^30–32^. ANN models assist in predictive modeling and optimization, while PSO fine-tunes ANN parameters, improving prediction accuracy and control. This approach aligns with recent advancements^33–35^ highlighting the innovative integration of optimization techniques in engineering and biomedical applications.

To improve future sensing devices, this study aims to optimize control parameters such as Reynolds number, Damköhler number, Schmidt number, and reaction surface position to reduce the response time of a microfluidic biosensor for SARS-CoV-2 detection. The design of experiments uses Taguchi’s L_9_ orthogonal array^36,37^, and machine learning models, specifically ANN-PSO, are developed to predict the microfluidic chip’s performance.

The originality and novelty of this work lie in using the Taguchi method combined with the PSO algorithm to predict biosensor performance for rapid SARS-CoV-2 detection. This approach offers potential advantages in efficiency, cost, and detection time. Unlike previous research focused on structural parameters^17–19,21^, our study prioritizes optimizing dimensionless numbers (Reynolds, Damköhler, and Schmidt numbers), providing a more comprehensive understanding and applicability across diverse microfluidic systems.

## Biochip design

**Fig. 1:**
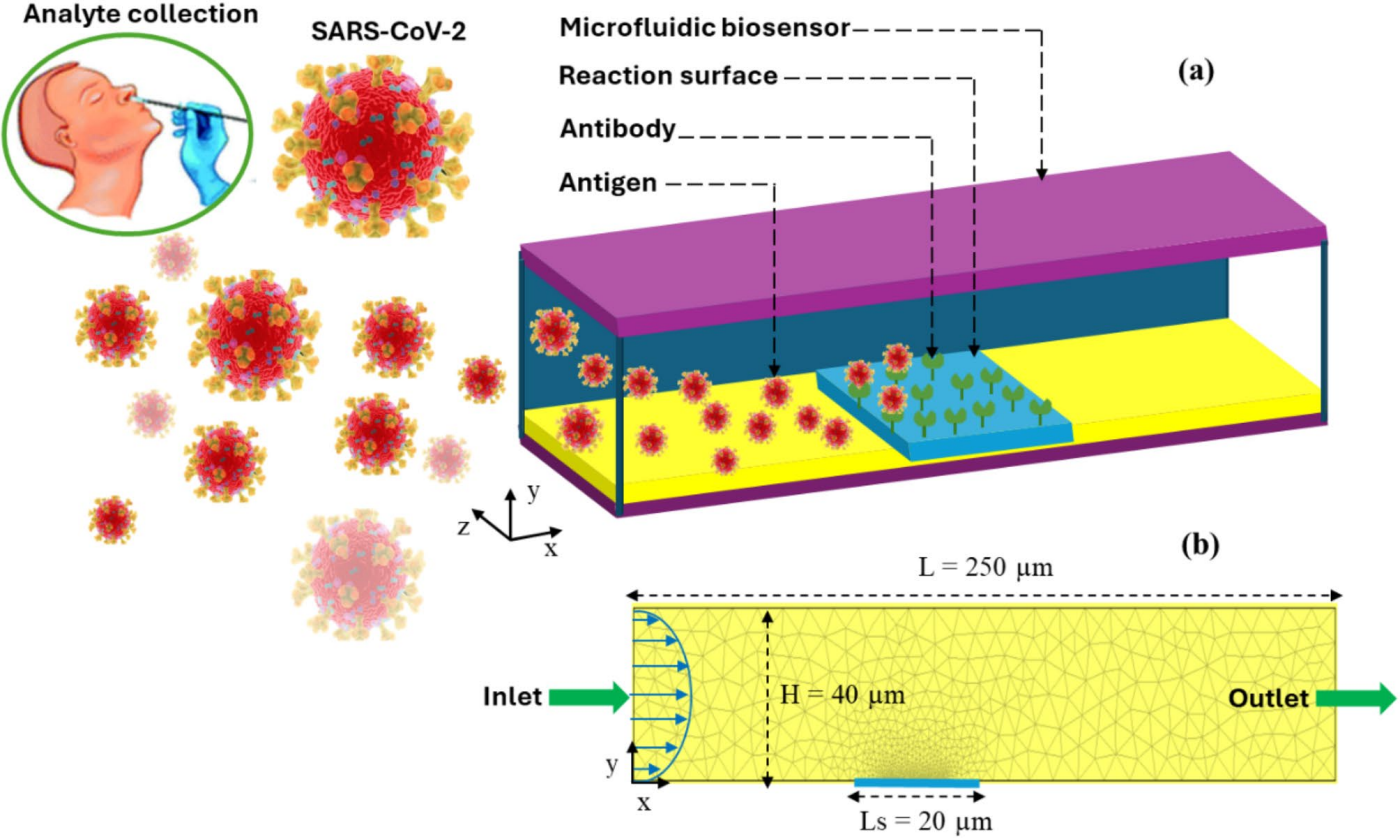
(**a**) Microfluidic biochip design. (**b**) 2D numerical domain. The 3D representation was generated using Microsoft PowerPoint 2016.

Figure 1a,b illustrates the geometry of the studied microfluidic chip. The microchannel has a length (L) of 250 μm and a height (H) of 40 μm. The reaction surface, measuring 20 μm, is situated on the bottom wall at a specific x position from the inlet of the microchannel. The carrier fluid, composed of water mixed with antigens (SARS-CoV-2), flows through the microchannel from left to right. Initially, ligands (antibodies) are immobilized on the reaction surface.

## Model equations

### Modeling Navier-Stokes equations

The fluid carrying antigens, assumed to be Newtonian and incompressible, flows in a laminar regime inside the microchannel. The continuity equation for incompressible fluids is:

1 $$\frac{\partial u}{\partial x}+\frac{\partial v}{\partial y} =0$$

Consequently, the 2D Navier-Stokes equations are employed to ascertain the fluid velocity field in the microchannel, as presented in Eqs. (2) and (3):

2 $$\rho \left(u\frac{\partial u}{\partial x}+v\frac{\partial u}{\partial y}\right)=-\frac{\partial p}{\partial x}+\mu \left(\frac{{\partial }^{2}u}{\partial {x}^{2}}+\frac{{\partial }^{2}u}{\partial {y}^{2}}\right)$$

3 $$\rho \left(u\frac{\partial v}{\partial x}+v\frac{\partial v}{\partial y}\right)=-\frac{\partial p}{\partial y}+\mu \left(\frac{{\partial }^{2}v}{\partial {x}^{2}}+\frac{{\partial }^{2}v}{\partial {y}^{2}}\right)$$

Here, *u* and *v* represent the components of the flow velocity field, while p, ρ, and µ denote the pressure, density, and dynamic viscosity of the fluid, respectively.

### Modeling the antigen transport equation

The diffusion-convection transport of the target antigen is modeled using Fick’s second law, as shown in Eq. (4):

4 $$\frac{\partial \left[A\right]}{\partial t}+u\frac{\partial \left[A\right]}{\partial x}+v\frac{\partial \left[A\right]}{\partial y}=D\left(\frac{{\partial }^{2}\left[A\right]}{\partial {x}^{2}}+\frac{{\partial }^{2}\left[A\right]}{\partial {y}^{2}}\right)$$

where [A] and D denote the concentration and the diffusion constant of the target antigen, respectively.

### Modeling the binding kinetics of the antigen-antibody reaction

Antigen molecules (analytes A) are transported by diffusion and convection to reach free binding sites (ligands B) immobilized on the sensitive surface. This process results in the formation of an analyte-ligand complex (AB), as described by the following reaction:

5 $$A+B\rightleftharpoons AB$$

According to the first-order Langmuir-Hinshelwood adsorption model^38^, the formation of the antigen-antibody complex (AB) is described by:

6 $$\frac{\partial \left[AB\right]}{\partial t}={k}_{on}\left[{A}_{surf}\right].\left[{B}_{free}\right]-{k}_{off}\left[AB\right]$$

where $\left[AB\right]$ represents the surface concentration of the complex, $\left[{A}_{surf}\right]$ is the volume analyte concentration at the binding surface, $\left[{B}_{free}\right]$ is the surface concentration of free ligands, ${k}_{on}$ is the complex association rate constant, and ${k}_{off}$ is the complex dissociation rate constant. The equilibrium of the reaction is described by the equilibrium dissociation constant ${K}_{d}=\frac{{k}_{off}}{{k}_{on}}$.

**Fig. 2:**
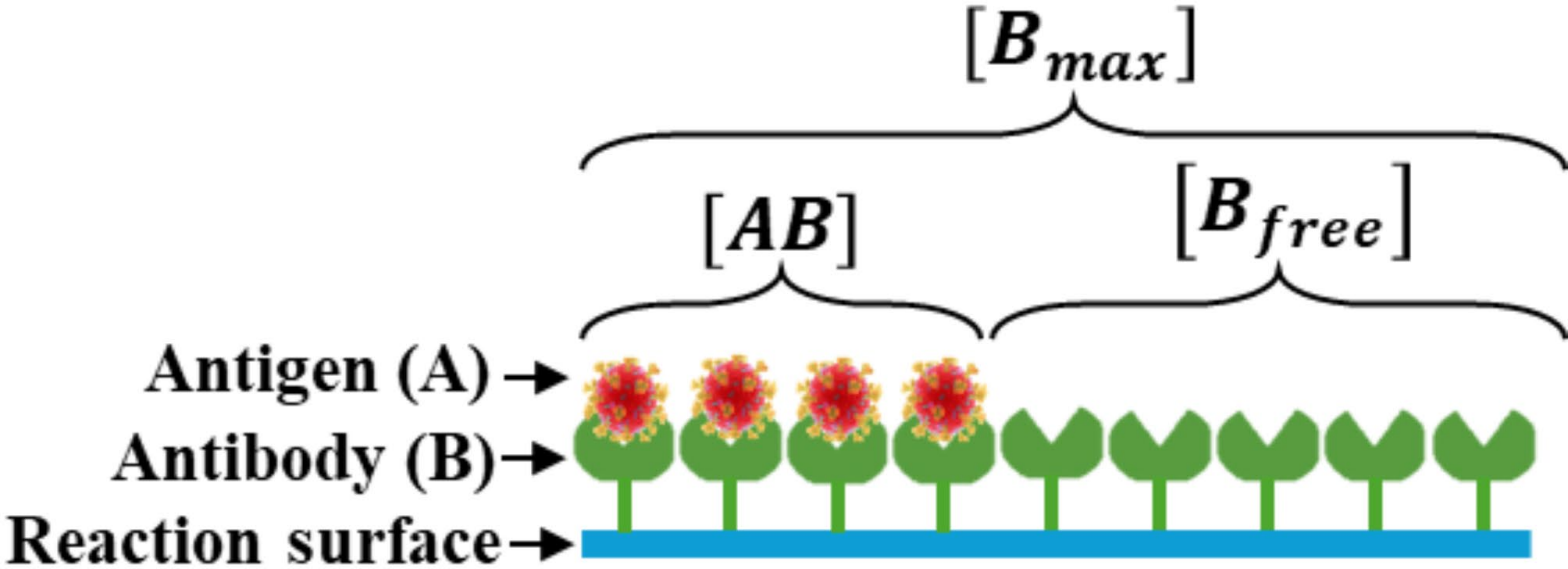
Antigen-antibody kinetic reaction.

As illustrated in Fig. 2, the concentration of available binding sites on the sensitive surface $\left[{B}_{max}\right]$ is equal to the sum of the concentrations of free binding sites $\left[{B}_{free}\right]$ and the bound complexes $\left[AB\right]$:

7 $$\left[{B}_{max}\right]=\left[{B}_{free}\right]+\left[AB\right]$$

The Eq. (6) is then written as (Eq. (8)):

8 $$\frac{\partial \left[AB\right]}{\partial t}={k}_{on}\left[{A}_{surf}\right].\left(\left[{B}_{max}-\left[AB\right]\right]\right)-{k}_{off}\left[AB\right]$$

## Dimensionless model equations

The model equations have been transformed into a dimensionless form as follows: The two components of the velocity vector are scaled by *u*_*0*_, which is the average velocity of the fluid at the inlet of the microchannel. The time, pressure and x and y coordinates are scaled by the diffusion transport time $\frac{{H}^{2}}{D}$, the pressure scale ρu_0_, and the length of the channel *H*, respectively. The surface concentration of the antigen-antibody complexes and the volume concentration of antigen molecules are scaled by the factors $\left[{B}_{max}\right]$ and $\left[{A}_{0}\right]$ respectively, where $\left[{A}_{0}\right]$ is the antigen concentration at the inlet of the microchannel.

The dimensionless form of Eqs. 1, 2, 3, 4 and 8 is then written as Eqs. 9, 10, 11, 12 and 13:

9 $$\frac{\partial {u}^{*}}{\partial {x}^{*}}+\frac{\partial {v}^{*}}{\partial {y}^{*}} =0$$

10 $${u}^{*}\frac{\partial {u}^{*}}{\partial {x}^{*}}+{v}^{*}\frac{\partial {u}^{*}}{\partial y}=-\frac{\partial {p}^{*}}{\partial {x}^{*}}+\frac{1}{Re}\left(\frac{{\partial }^{2}{u}^{*}}{\partial {{x}^{*}}^{2}}+\frac{{\partial }^{2}{u}^{*}}{\partial {{y}^{*}}^{2}}\right)$$

11 $${u}^{*}\frac{\partial {v}^{*}}{\partial {x}^{*}}+v\frac{\partial {v}^{*}}{\partial {y}^{*}}=-\frac{\partial {p}^{*}}{\partial {y}^{*}}+\frac{1}{Re}\left(\frac{{\partial }^{2}{v}^{*}}{\partial {{x}^{*}}^{2}}+\frac{{\partial }^{2}{v}^{*}}{\partial {{y}^{*}}^{2}}\right)$$

12 $$\frac{\partial {\left[A\right]}^{*}}{\partial {t}^{*}}+Pe\left({u}^{*}\frac{\partial {\left[A\right]}^{*}}{\partial {x}^{*}}+{v}^{*}\frac{\partial {\left[A\right]}^{*}}{\partial {y}^{*}}\right)=\frac{{\partial }^{2}{\left[A\right]}^{*}}{\partial {{x}^{*}}^{2}}+\frac{{\partial }^{2}{\left[A\right]}^{*}}{\partial {{y}^{*}}^{2}}$$

13 $$\frac{\partial {\left[AB\right]}^{*}}{\partial {t}^{*}}=Da.\sigma \left[{\left[{A}_{surf}\right]}^{*}.\left(\left[1-{\left[AB\right]}^{*}\right]\right)-{K}_{d}{\left[AB\right]}^{*}\right]$$

where

$${u}^{*}=\frac{u}{{u}_{0}}, {v}^{*}=\frac{v}{{v}_{0}}, {x}^{*}=\frac{x}{H}, {y}^{*}=\frac{y}{H}, {t}^{*}=\frac{t}{{t}_{0}}, {\left[A\right]}^{*}=\frac{\left[A\right]}{\left[{A}_{0}\right]}, {\text{t}}_{0}=\frac{{\text{H}}^{2}}{\text{D}},{p}^{*}=\frac{p}{{p}_{0}}, {p}_{0}=\frac{1}{2} \rho {u}_{0}^{2}$$

where $Re=\frac{\rho {u}_{0}H}{\mu }$ represents the Reynolds number, $Pe=\frac{{u}_{0}H}{D}$ is the Peclet number, $Da=\frac{{K}_{on}\left[{B}_{max}\right]H}{D}$ is the Damkohler number, $\sigma =\frac{H\left[{A}_{0}\right]}{\left[{B}_{max}\right]}$ is the relative density of the analyte-ligand complex, and ${K}_{d}=\frac{{K}_{off}}{{K}_{on}\left[{A}_{0}\right]}$ is the dimensionless equilibrium dissociation constant. The Damkohler number, used in chemical kinetics, defines the ratio between the characteristic diffusion time of the antigen (rate of the transport $\frac{D}{H}$) and the characteristic reaction time for complex formation (reaction velocity ${K}_{on}.\left[{B}_{max}\right]$).

Da > 1 indicates a transport-limited system, whereas Da < 1 specifies a reaction- rate-limited system. In the case of large molecules, such as the analyte in this study, Da will be large due to the small diffusion coefficient^39^.

The detection time of the microfluidic biosensor is a crucial parameter of the analyte-ligand chemical kinetics, representing the duration required for the concentration of the analyte-ligand complex to reach 95% of its threshold value. Here the spatially averaged dimensionless concentration of AB complexes is defined as:

14 $$\langle\left[AB\right]\rangle=\frac{1}{{l}_{s}}\underset{0}{\overset{{l}_{s}}{\int }}\left[AB\right]\left(x,t\right)dx$$

where ${\varvec{l}}_{\varvec{s}}=\frac{{l}_{s}}{H}$ is the dimensionless length of the binding surface.

## Boundary and initial conditions

For the modeling of the laminar flow, at the inlet of the microfluidic channel, the fluid flows with a parabolic velocity profile of average value *u*_*0*_. At the outlet, the flow is assumed to be fully developed. For the lower and upper microchannel walls, including the reaction surface, the no-slip condition is applied. Regarding the modeling of antigen transport, a constant volume concentration ${\left[A\right]}_{0}$ and a convective flow condition, $\overrightarrow{n}.\left(D\nabla \left[A\right]\right)=0$, were imposed at the inlet and outlet of the microchannel, respectively. For the reaction surface, the condition of diffusive flux balanced by the temporal flow rate was applied. For the rest of the microchannel walls, they are assumed to be impermeable (i.e., they do not interact with the target antigens) and the homogeneous Neumann condition was adopted^17,21^.

For the initial conditions concerning Eqs. (12) and (13), the analyte and surface complex concentrations were initially set to zero: ${\left[\varvec{A}\right]}_{(t=0)}=0$ and ${\left[\varvec{A}\varvec{B}\right]}_{(t=0)}=0$.

## Simulations protocols

**Fig. 3:**
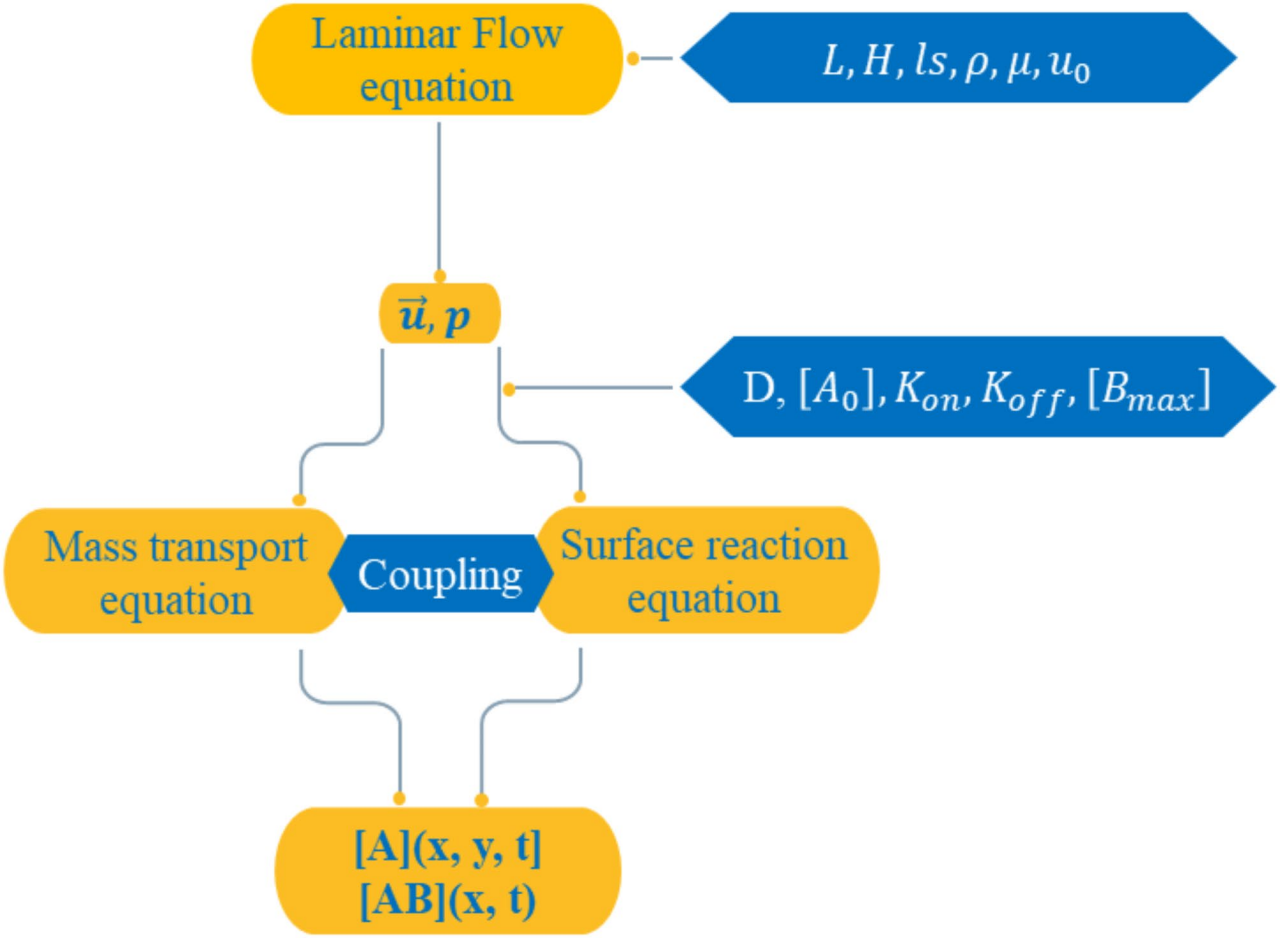
Algorithm of numerical simulation protocols.

To solve the model comprising Eqs. (9–13), the finite element method (FEM) was employed^40^. The 2D domain is divided into triangular cells, with mesh refinement near the sensitive surface. The numerical resolution process is shown in Fig. 3. The transport equations, coupled with the first Langmuir adsorption model, are solved using the finite element method (FEM) with the Galerkin approach. A computer code has been developed to compute the numerical solution^41^. First, triangular elements are used to discretize the domain and the mesh is refined near the reaction surface and electrodes to improve the quality. All variables are then approximated by polynomials within each element. Subsequently, the concentrations of the target antigen, [A] (x, y, t), in the microchannel and the antigen-antibody complex, [AB] (x, t), on the sensitive surface were obtained by simultaneously solving the antigen transport and the binding reaction equations in a time-dependent regime. In this study, the target antigen is the SARS-CoV-2 virus, and the ligand is its corresponding antibody (b1 or h12)^21^. To compute the total concentration of the formed complexes (SARS-CoV-2-antibody), the local concentration was integrated over the entire length of the binding surface (Eq. 14) and the normalized surface concentration of these complexes, $\left[\overline{AB}\right]$, was then calculated by dividing the total concentration by the concentration of binding sites on the biosensor surface, $\left[{B}_{max}\right]$.

**Fig. 4:**
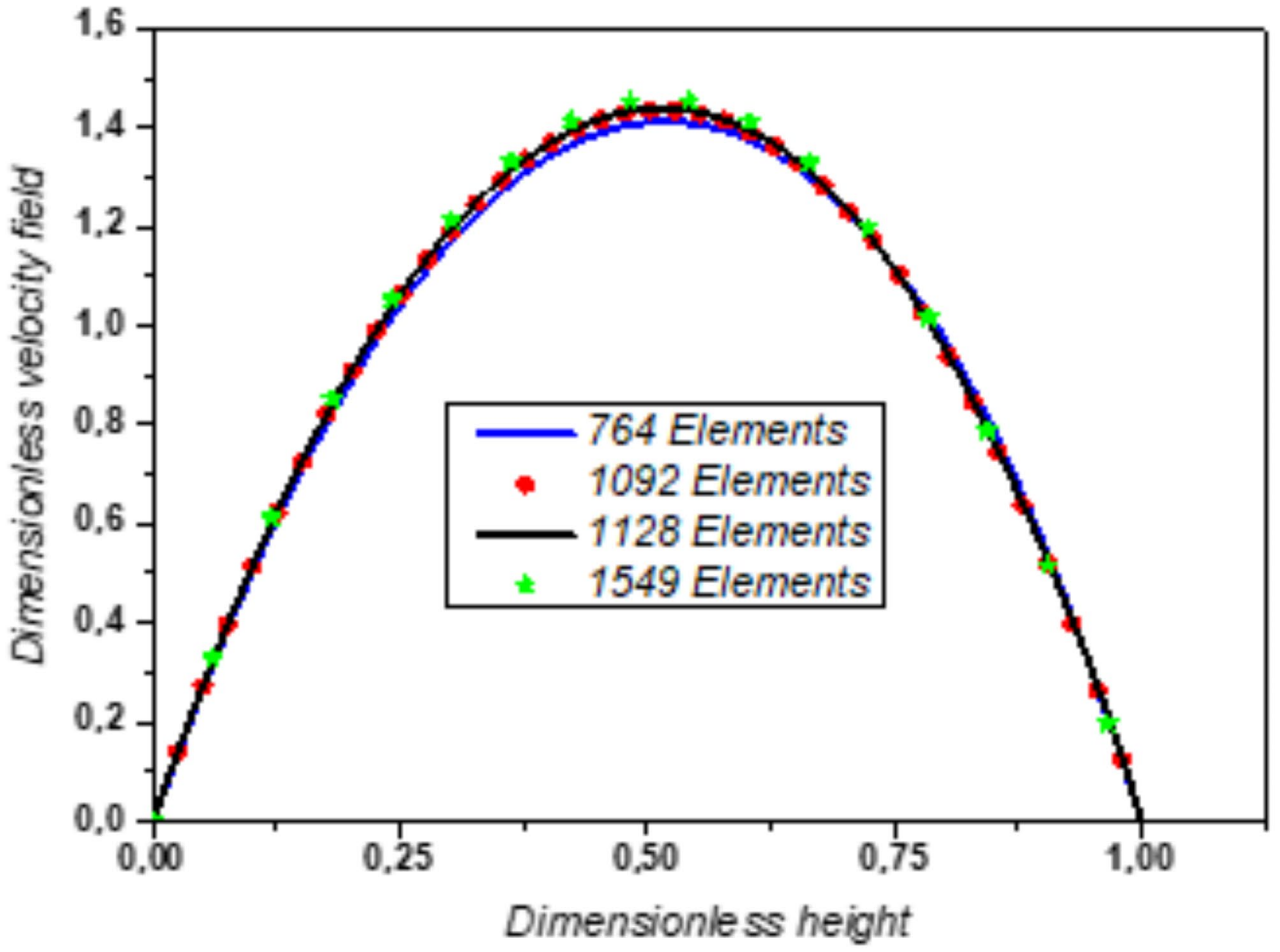
Dimensionless velocity profile along the y-axis at dimensionless **x** = 2 for different mesh grids.

Grid independence was assessed through a mesh sensitivity analysis of the dimensionless velocity profile along the y-axis at dimensionless **x** = 2 for several meshes (764, 1092, 1128 and 1549 elements) as shown in Fig. 4.

The error results show that the relative variation of successive values of u at each mesh point is calculated using the following expression:

$$Erro{r}_{j}=\left|\frac{{u}_{i+1}-{u}_{i}}{{u}_{i}}\right|\times 100$$

**Fig. 5:**
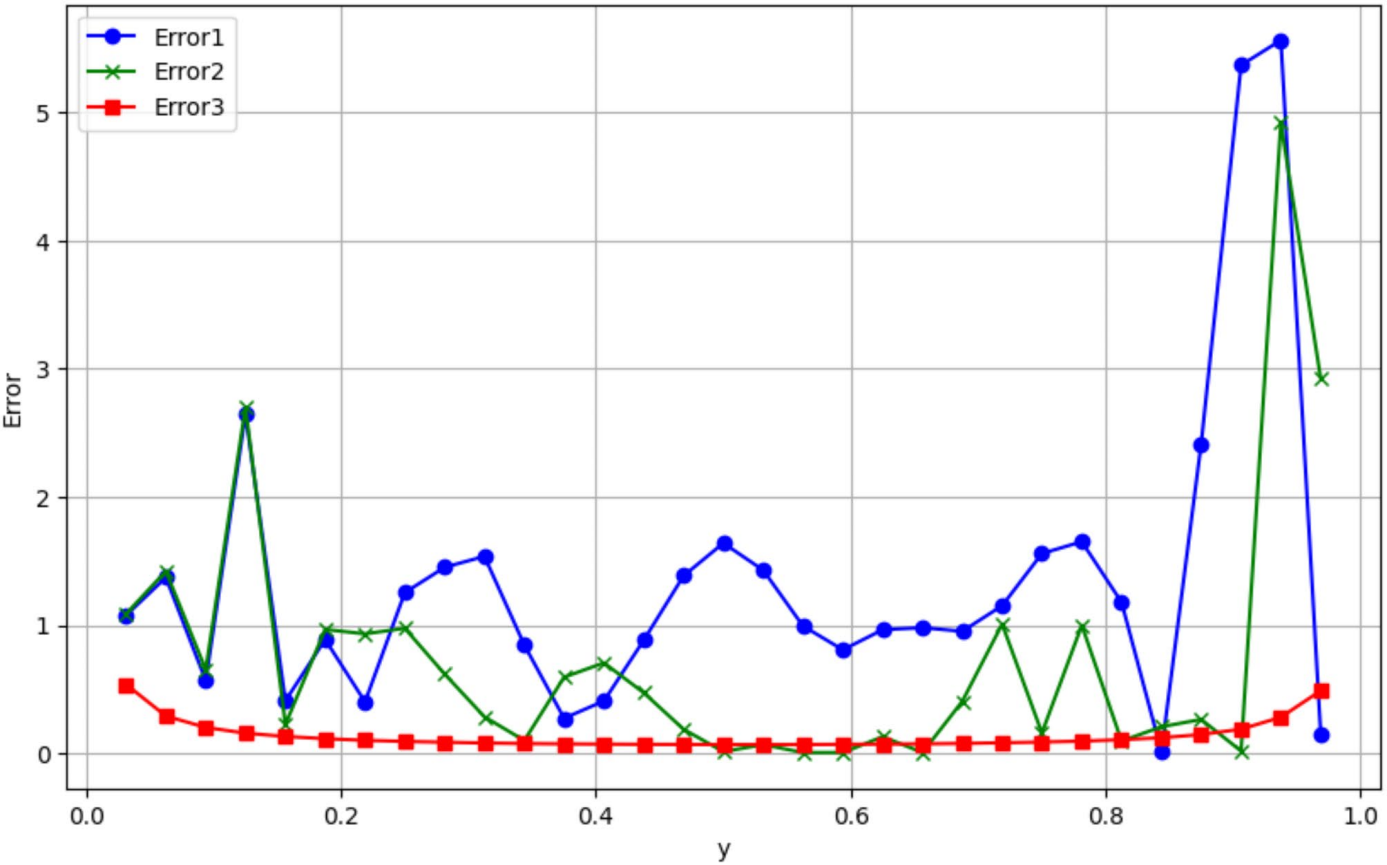
Distribution of relative error along the y-axis for different meshes.

For other values of x, the fluctuating errors indicate more or less significant variations in u(i). For example, for x = 0.0313, the error for Error1 and Error2 is about 1%, while Error3 has a much smaller error (0.54%) (Fig. 5). This indicates that the successive variations of u are larger for Error1 and Error2 and more stable for Error3. In summary, the error peaks correspond to abrupt changes in u, while the smaller errors indicate a more regular variation of u. In conclusion, since the error of mesh 2 compared to mesh 1 remains less than 5%, mesh 2 (composed of 1092 elements) is retained for this optimization study.

## Results and discussion

### Model validation

**Fig. 6:**
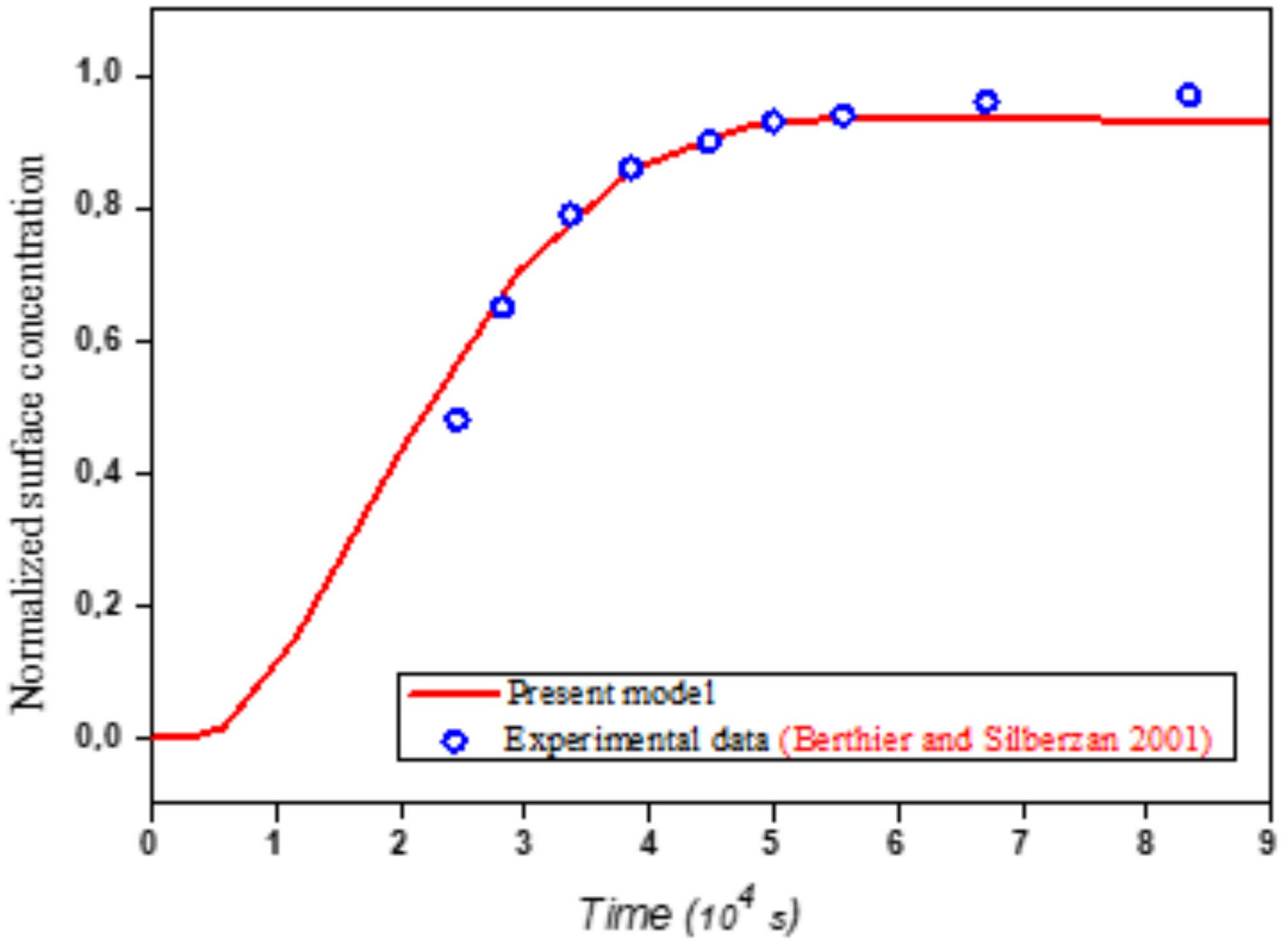
Comparison of our model with the experimental data from Berthier and Silberzan^21^.

First, the numerical model was validated by comparing it with the experimental data of Berthier and Silberzan^21^, as shown in Fig. 6. The time-normalized surface concentration during the adsorption phase was calculated using the same experimental parameters within a microfluidic channel measuring 1 mm in height and 1 cm in width. The target antigens’ concentration and their diffusion constant are 2.5 × 10^− 6^ Mol/m^3^ and 7 × 10^− 11^ m^2^/s, respectively. The flow rate of the carrier fluid is 10^− 6^ m^3^/s. The density of binding sites, the association constant, and the dissociation constant are 1.668 × 10^− 8^ Mol/m^2^, 75 m^3^/Mol⋅s and 10^− 2^ 1/s, respectively.

The following coefficients, namely the Root Mean Square Error (RMSE) and the Mean Absolute Percentage Error (MAPE), are calculated to evaluate the performance of the model proposed in this study^42,43^.

15 $$RMSE=\sqrt{\frac{1}{N}\sum _{i=1}^{N}{\left({y}_{i}-{\widehat{y}}_{i}\right)}^{2}}$$

16 $$MAPE=\frac{1}{N}\sum _{i=1}^{N}\left|\frac{{y}_{i}-{\widehat{y}}_{i}}{{y}_{i}}\right|\times 100$$

where $y$ is the actual value obtained by the experimental data and $\widehat{y}$ is the predicted value using one of the model and N and are numbers of observations.

The calculated Mean Absolute Error (MAE) of 0.0180 and Root Mean Square Error (RMSE) of 0.0298 indicate that the model’s predictions are quite accurate. The small MAE suggests that, on average, the model’s predictions deviate by only 0.0180 units from the experimental data, while the slightly higher RMSE reflects some larger deviations. Overall, the model demonstrates good performance in predicting the response with minimal error.

### 2D approximation for microfluidic biosensor analysis

**Fig. 7:**
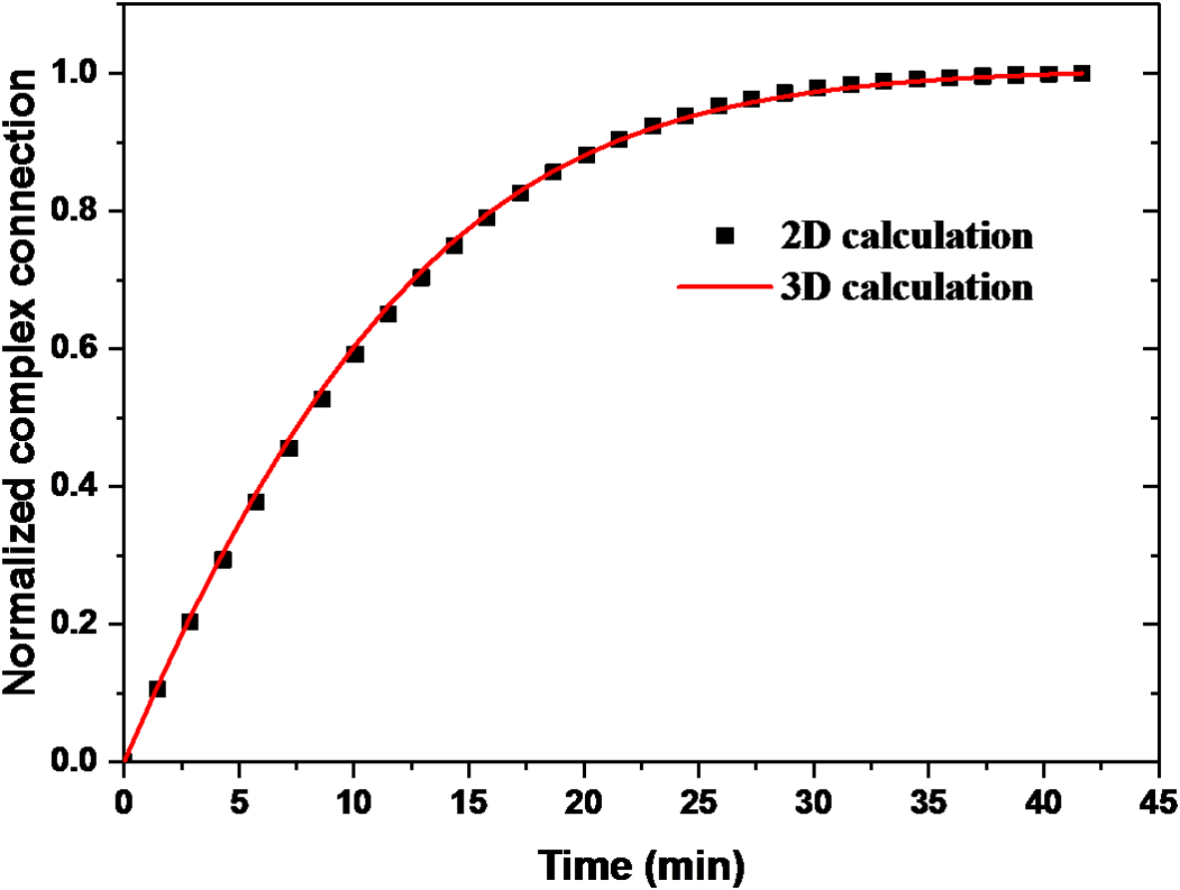
Comparison between 3D and 2D calculation.

The graph of Fig. 7 shows the evolution of the normalized concentration of the complex as a function of time for both the 2D and 3D models. The results show a slight underprediction bias with a mean error of -0.002476. The errors are consistent, as indicated by the low standard deviation of 0.006587112, and the maximum error is small at 0.00513, indicating good overall accuracy and stability.

This similarity shows that the 2D approximation faithfully reproduces the results of the 3D model, justifying its use for the study of the microfluidic biosensor while preferentially reducing the computational cost.

### Parameters and levels selection

To evaluate the influence of control parameters on SARS-CoV-2 binding kinetics, we performed numerical simulations using the geometric design of the microfluidic biosensor shown in Fig. 1. The simulation parameters employed in this study include the input volume concentration of the SARS-CoV-2 antigen ($\left[{A}_{0}\right]={10}^{-7}Mol.{m}^{-3}$), the antibody concentration on the sensitive surface ($\left[{B}_{max}\right]=3.3.{10}^{-8}Mol.{m}^{-2}$), and the equilibrium desorption constant (${K}_{d}={10}^{-6}Mol.{m}^{-3}$), which align with values reported in the literature^17–19,21,44^. Consequently, the relative density of the analyte-ligand complex, σ, is determined to 1.2.10^−4^. In this study, different controllable parameters were chosen for optimization to maximize the biosensor’s efficiency. The Reynolds number (Re), Damkohler number (Da), Schmidt number (Sc), and the position of the reaction surface (X) are the four variables targeted for optimization using the Taguchi method, with the objective of achieving the shortest detection time.

#### Reynolds number

In microfluidic devices, flow velocities typically remain low, ranging from a few tenths of a millimeter per second. For water as the carrier fluid ($\rho ={10}^{3}\,{\text{kg}}\,{m}^{-3}, \mu =1.08\times{10}^{-3}{\text{Pa\,s}})$, the Reynolds number varies between 4.10^− 3^ and 4.10^− 2^ for flow velocities ranging from 10^− 4^ to 10^− 3^ m.s^− 1^^45,46^.

#### Damkohler number

With the surface concentration of binding sites fixed ($\left[{B}_{max}\right]=3,3\times{10}^{-8}\,{\text{mol\,m}}^{-2}$) and the microchannel height $(H=40.{10}^{-6}m)$, the Damkohler number is influenced solely by the diffusion coefficient of the target antigen (D) and the adsorption antigen-antibody constant (${K}_{on}$). For SARS-CoV-2, with the diffusion coefficient ranging from 10^−11^ to 10^−10^ m^2^. s^−1^ and the adsorption constant between 10^2^ and 10^4^ m^3^/Mol.s, the Damkohler number can fluctuate between 1 and 1000^10,47,48^.

#### Schmidt number

Given that the Schmidt number ($Sc=\frac{Pe}{Re})$ is inversely proportional to the antigen diffusion coefficient (D) for a given density and dynamic viscosity of the carrier fluid, it can vary between 10^4^ and 10^5^ for the same range of D variation indicated previously^19^.

### Taguchi method optimization

The Taguchi method was employed in this numerical simulation to streamline the testing process for achieving the shortest detection time in the microfluidic biosensor. The selection of the Taguchi Design of Experiments methodology is justified by its systematic and efficient approach to optimizing process parameters. The Taguchi method allows for a reduction in the number of experiments required to determine optimal conditions, which is particularly useful in complex systems like microfluidic biosensors^49^.

Table 1 outlines the four factors influencing the detection system, each with three levels denoted as “1,” “2,” and “3,” representing the lowest, mid, and highest levels, respectively. Considering these factors (A, B, C, and D), conducting experiments for all possible combinations would require 3^4^ = 81 trials. To minimize the number of experiments, the Taguchi method was applied using the orthogonal table L_9_(3^4^), as presented in Table 2. This approach reduced the number of experiments to nine, involving four critical parameters at three levels each, without considering their interactions.

**Table 1:** Selected optimization factors and respective levels.

Symbol	Optimization parameter	Level 1	Level 2	Level 3
*A*	Reynolds number (Re)	4.10^− 3^	2.10^− 2^	4.10^− 2^
*B*	Damkohler number (Da)	5	500	1000
*C*	Schmidt number (Sc)	10^4^	5.10^4^	10^5^
*D*	Reaction surface position (X)	1	2.5	4

**Table 2:** The Taguchi L_9_(3^4^) orthogonal table.

Experiment tests	Factors levels			
	A	B	C	D
1	1	1	1	1
2	1	2	2	2
3	1	3	3	3
4	2	1	2	3
5	2	2	3	1
6	2	3	1	2
7	3	1	3	2
8	3	2	1	3
9	3	3	2	1

**Fig. 8:**
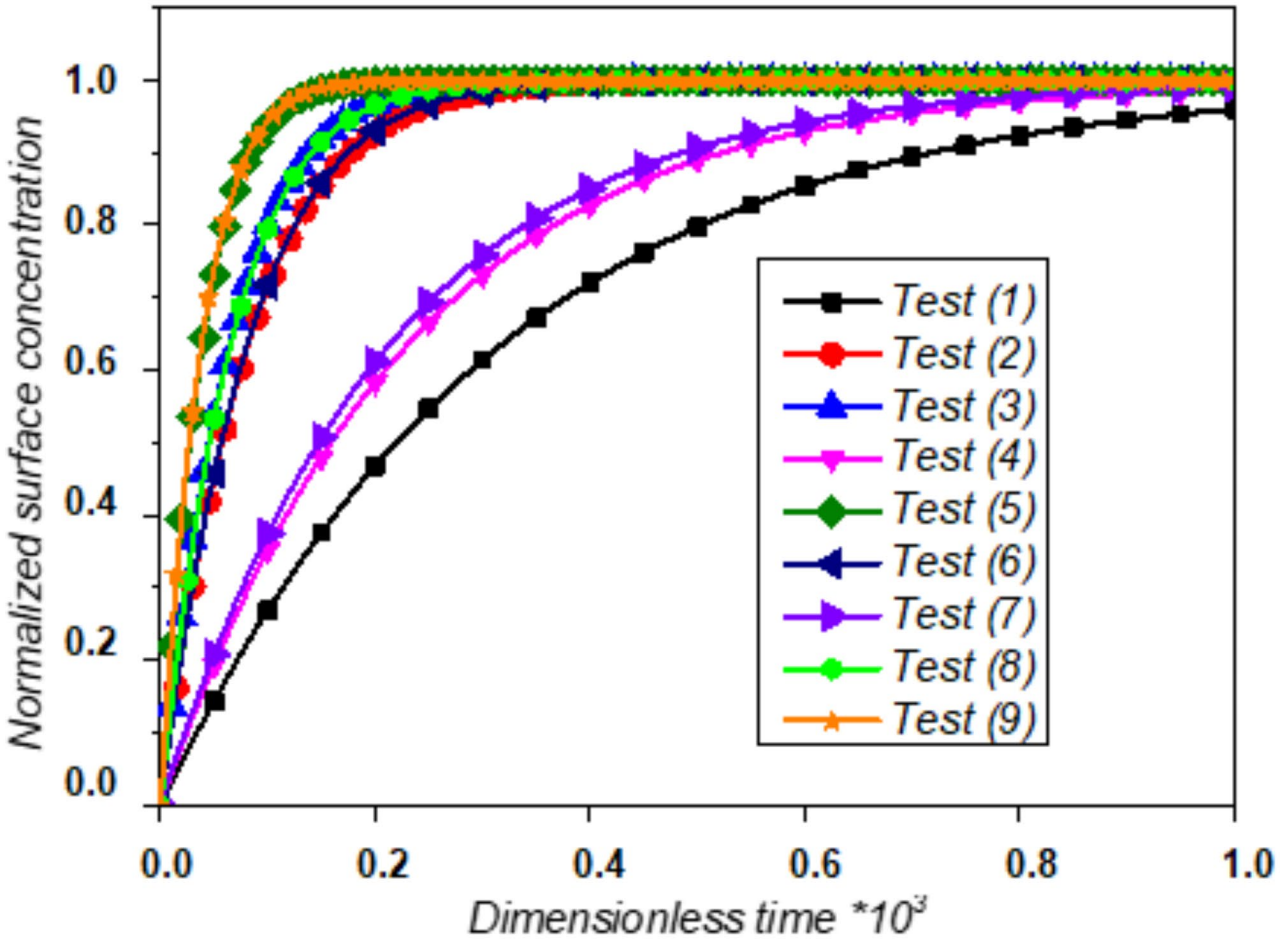
Normalized complex concentration versus time for the nine Taguchi tests.

In each test, the factors are set at levels 1, 2, or 3. Figure 8 illustrates the average normalized dimensionless concentration of the antigen-antibody complex over dimensionless time for all experimental tests conducted according to Table 2.

In the Taguchi design of experiments, we manipulate noise factors to intentionally introduce variability. From the obtained results, we can identify optimal parameters that make the detection process robust against variations caused by these noise factors. A high signal-to-noise ratio (S/N) value indicates that the control factor parameters effectively limit the effects of the noise factors. The S/N ratio, calculated based on the “smaller is better” criteria for each test, is determined using Eq. (15)^50^, and the results are recorded in Table 3.

17 $$S/N=-10{log}\left(\frac{1}{n}\sum _{i=1}^{n}{Y}_{i}^{2}\right)$$

**Table 3:** The Taguchi L_9_(3^4^) designation with four factors at three levels, detection time, and S/N ratio obtained from the corresponding runs.

Experimental run	Designation	Dimensionless Response time (T_*R*_)	S/*N* ratio for T_*R*_
1	A_1_B_1_C_1_D_1_	930	− 59,3697
2	A_1_B_2_C_2_D_2_	225	− 47,0437
3	A_1_B_3_C_3_D_3_	175	− 44,8608
4	A_2_B_1_C_2_D_3_	680	− 56,6502
5	A_2_B_2_C_3_D_1_	105	− 40,4238
6	A_2_B_3_C_1_D_2_	220	− 46,8485
7	A_3_B_1_C_3_D_2_	630	− 55,9868
8	A_3_B_2_C_1_D_3_	170	− 44,6090
9	A_3_B_3_C_2_D_1_	100	− 40,0000

Here, S represents the signal value, N is the noise value, n is the number of simulation tests, and y_i_ is the measured response value (detection time of the i^th^ simulation). A higher S/N value indicates better performance^50^, and the optimal level of each parameter is specified by a higher S/N values. Table 3 shows the numerical results for the biosensor’s response time (T_R_) and its corresponding S/N ratio using the experimental layout.

**Fig. 9:**
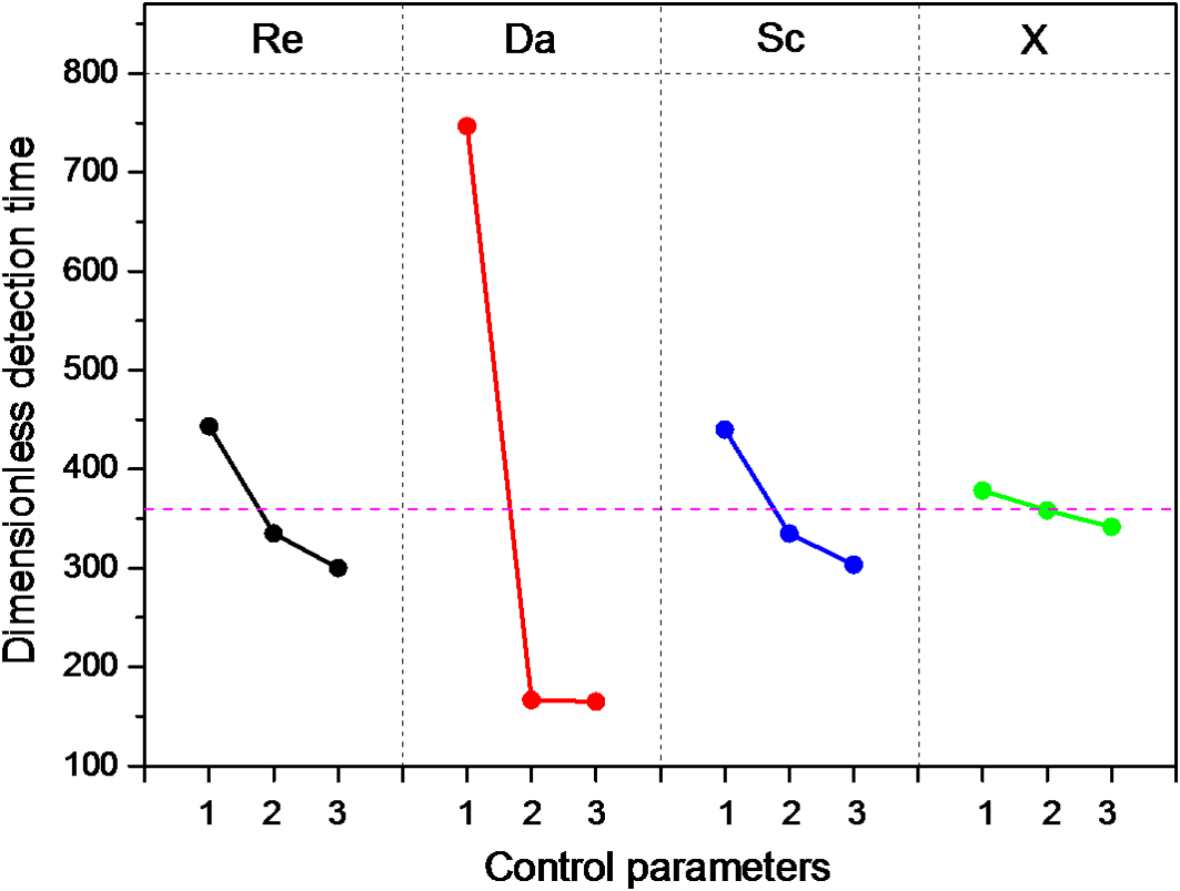
Main effect plots of each key parameter on the detection time of the device.

To evaluate the impact of each key parameter, it is essential to calculate the mean values of the responses for each level. This involves summing the results associated with each level in the orthogonal table and dividing by the number of tests for that level to obtain the appropriate averages. Figure 9 visually represents the average effects of the four factors considered in this study. The factor with the most substantial influence is identified by the difference values (Delta) between the maximum and minimum values of the three averages. The greater the difference, the more influential the control factor. In Fig. 9, the Damkohler number stands out as having the strongest influence.

The significance of each key parameter is further analyzed in Table 4 by subtracting the maximum S/N ratio from its minimum value across the three levels. Parameters with minimal differences in the S/N ratio play a smaller role in controlling the synthesis process^37^.

**Table 4:** Signal to noise ratios for each level.

Level	Re	Da	Sc	X
1	− 50,42	− 57,34	− 50,28	− 46,60
2	− 47,97	− 44,03	− 47,90	− 49,96
3	− 46,87	− 43,90	− 47,09	− 48,71
Delta = Max-Min	3,56	13,43	3,19	3,36
Rank	2	1	4	3

The Schmidt number (Sc) plays a key role in antigen diffusion and, consequently, on the detection time. Being defined as the ratio of momentum diffusivity to mass diffusivity (Sc = ν/D), a high Sc implies a lower antigen diffusivity, which can prolong the detection time. Conversely, a lower Sc favors diffusion and can thus improve the detection efficiency. In order to better answer this question, we will add an in-depth discussion supported by numerical results to illustrate the impact of Sc variation on antigen transport and detection performance. In particular, we will analyze the evolution of antigen concentration as a function of time for different Sc values, thus highlighting its effect on the sensor efficiency^51^.

**Fig. 10:**
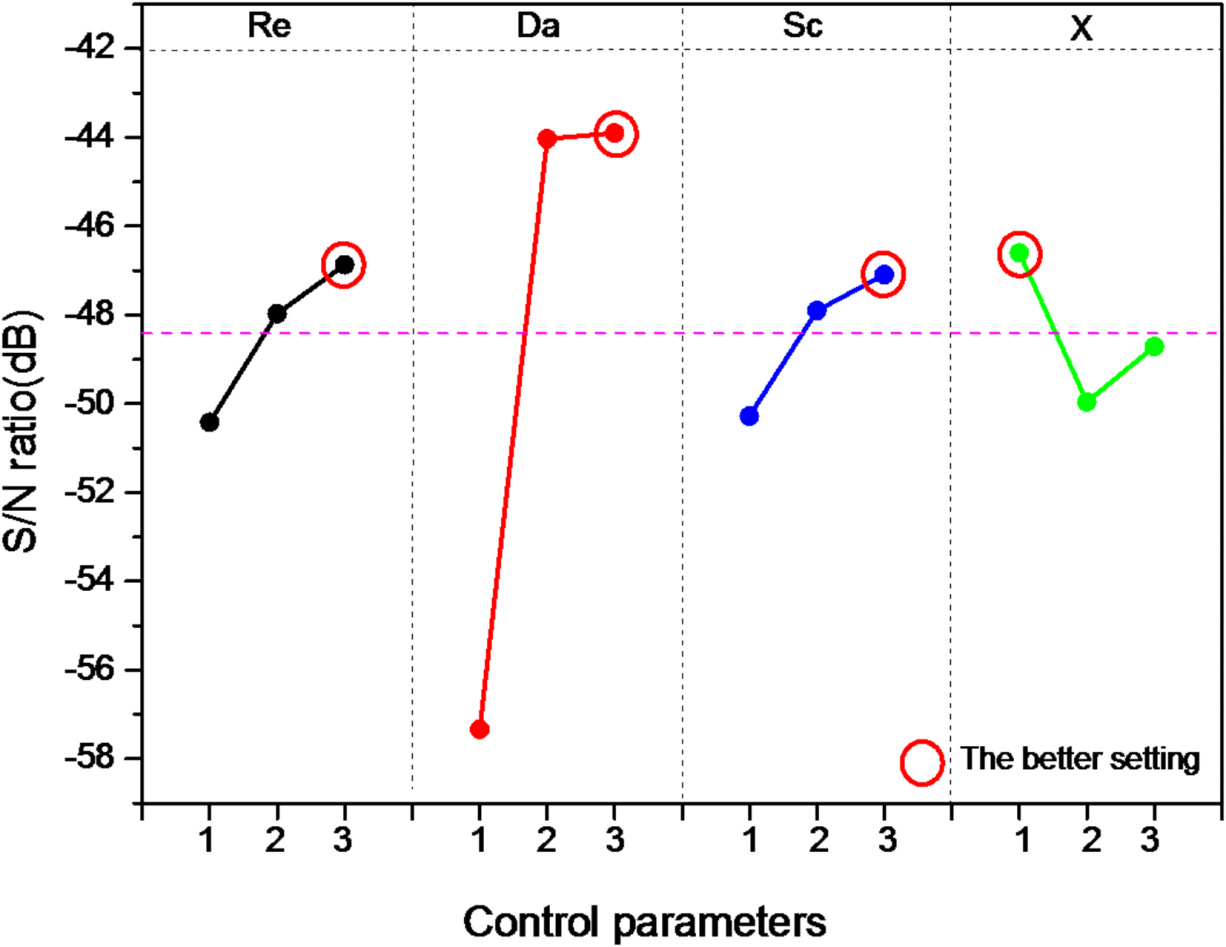
S/N ratio for the four key parameters at different levels.

Plotting the S/N ratio against each key parameters, as per the values in Table 4, reveals in Fig. 10 that, according to the Taguchi method, the lowest value of the biochip’s response time is reached at the highest levels of the Reynolds number (4 × 10^− 2^), Damkohler number (10^3^), Schmidt number (10^5^), and the lowest level of the reaction surface position (1). Interestingly, the optimal combination (A_3_B_3_C_3_D_1_) was not among the nine tests of the L_9_ orthogonal network, but Taguchi’s method successfully identified it.

To justify the robustness of the optimization process with the Taguchi method and ensure the reliability of the S/N ratios and ANOVA results, we performed a new simulation of the biosensor’s response time using the optimized parameters. This validation procedure yielded a dimensionless detection time value of 80 (~ 21 min), the lowest among those obtained in the original L_9_ experiments, thus confirming the effectiveness of the optimization approach. Under optimal conditions, the limit of detection (LOD) and the limit of quantification (LOQ) were calculated 2.197 pmol/L and 6.659 pmol/L respectively.

### ANOVA analysis

Following the ANOVA scheme used for the L_9_ Taguchi method^52^, the percentage contribution of each key parameter to the detection time is determined in this work. The equations used in this analysis are as follows (Eqs. 16, 17, 18, 19, 20):

- The average of all response times (${\overline{\text{T}}}_{{\text{R}}}$) is calculated as:
  18 $${\overline{\text{T}}}_{{\text{R}}}=\frac{1}{9}\sum _{{\text{i}}=1}^{9}{\text{T}}_{{\text{R}}_{{\text{i}}}}$$
- The total sum of squares (${\text{SS}}_{{\text{Total}}}$) is determined by:
  19 $${\text{SS}}_{{\text{Total}}}=\sum _{{\text{i}}=1}^{9}{({\text{T}}_{{\text{R}}_{{\text{i}}}}-{\overline{\text{T}}}_{{\text{R}}})}^{2}$$
- The sum of squares for Reynolds number (${\text{SS}}_{{\text{Re}}}$), Damkohler number (${\text{SS}}_{{\text{Da}}}$), Schmidt number (${\text{SS}}_{{\text{Sc}}}$) and reaction surface position (${\text{SS}}_{{\text{X}}}$) are given by:
  20 $$\begin{aligned} {\text{SS}}_{{{\text{Re}}}} & = 3\sum\limits_{{{\text{i}} = 1}}^{3} {({\text{T}}_{{{\text{R}}_{{{\text{Rei}}}} }} - \overline{{\text{T}}} _{{\text{R}}} )^{2} } ,\quad {\text{SS}}_{{{\text{Da}}}} = 3\sum\limits_{{{\text{i}} = 1}}^{3} {({\text{T}}_{{{\text{R}}_{{{\text{Dai}}}} }} - \overline{{\text{T}}} _{{\text{R}}} )^{2} } ,\quad {\text{SS}}_{{{\text{Sc}}}} = 3\sum\limits_{{{\text{i}} = 1}}^{3} {({\text{T}}_{{{\text{R}}_{{{\text{SCi}}}} }} - \overline{{\text{T}}} _{{\text{R}}} )^{2} } \quad {\text{and}} \\ {\text{SS}}_{{\text{X}}} & = 3\sum\limits_{{{\text{i}} = 1}}^{3} {({\text{T}}_{{{\text{R}}_{{{\text{Xi}}}} }} - \overline{{\text{T}}} _{{\text{R}}} )^{2} } \\ \end{aligned}$$
  where ${\text{T}}_{{{\text{R}}_{\text{x}}}_{\text{i}}}$ is the i-th average response time of the corresponding parameter x (Re, Da, Sc, X) in the Taguchi design.
- the mean squares for each parameter are:
  21 $${\text{MS}}_{{{\text{Re}}}} = \frac{{{\text{SS}}_{{{\text{Re}}}} }}{{{\text{DF}}_{{{\text{Re}}}} }},\quad {\text{MS}}_{{{\text{Da}}}} = \frac{{{\text{SS}}_{{{\text{Da}}}} }}{{{\text{DF}}_{{{\text{Da}}}} }},\quad {\text{MS}}_{{{\text{Sc}}}} = \frac{{{\text{SS}}_{{{\text{Sc}}}} }}{{{\text{DF}}_{{{\text{Sc}}}} }}\quad {\text{and}}\quad {\text{MS}}_{{\text{X}}} = \frac{{{\text{SS}}_{{\text{X}}} }}{{{\text{DF}}_{{\text{X}}} }}$$
  where DF is the degree of freedom (2 = number of level − 1).
- The contribution percentages for each parameter are:
  22 $$\begin{aligned} {\text{\% }}\,{\text{contribution}}\,{\text{Re}} & = \frac{{{\text{SS}}_{{{\text{Re}}}} }}{{{\text{SS}}_{{{\text{Total}}}} }},\quad {\text{\% }}\,{\text{contribution}}\,{\text{Da}} = \frac{{{\text{SS}}_{{{\text{Da}}}} }}{{{\text{SS}}_{{{\text{Total}}}} }},\quad {\text{\% }}\,{\text{contribution}}\,{\text{Sc}} = \frac{{{\text{SS}}_{{{\text{Sc}}}} }}{{{\text{SS}}_{{{\text{Total}}}} }}\quad {\text{and}} \\ {\text{\% }}\,{\text{contribution}}\,{\text{X}} & = \frac{{{\text{SS}}_{{\text{X}}} }}{{{\text{SS}}_{{{\text{Total}}}} }} \\ \end{aligned}$$

**Fig. 11:**
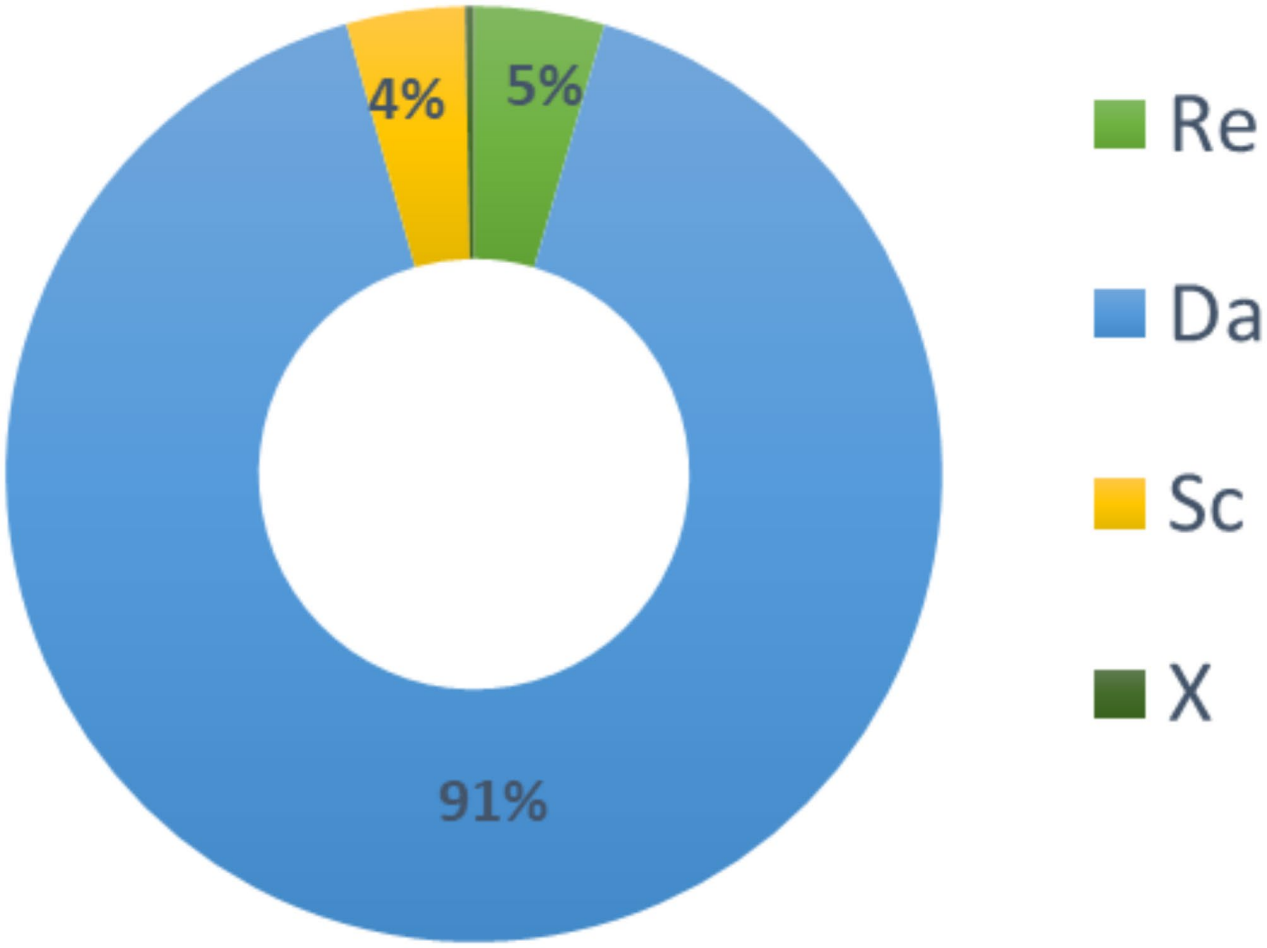
Contributions of key parameters to biosensor detection time.

The obtained results are presented in Table 5; Fig. 11. Among the key parameters, the Damkohler number (Da) has the highest contribution (91.1%) to reducing the response time of the device, while the reaction surface position (X) has the lowest contribution (0.3%).

**Table 5:** ANOVA results on the detection time response.

Source	DF	SS	MS	% Contribution
Re	2	33,506	16,753	4.5
Da	2	674,739	337,369	91.1
Sc	2	30,706	15,353	4.1
X	2	2022	1011	0.3
Residual error	0	0	0	0
Total	8	740,972		100

**Fig. 12:**
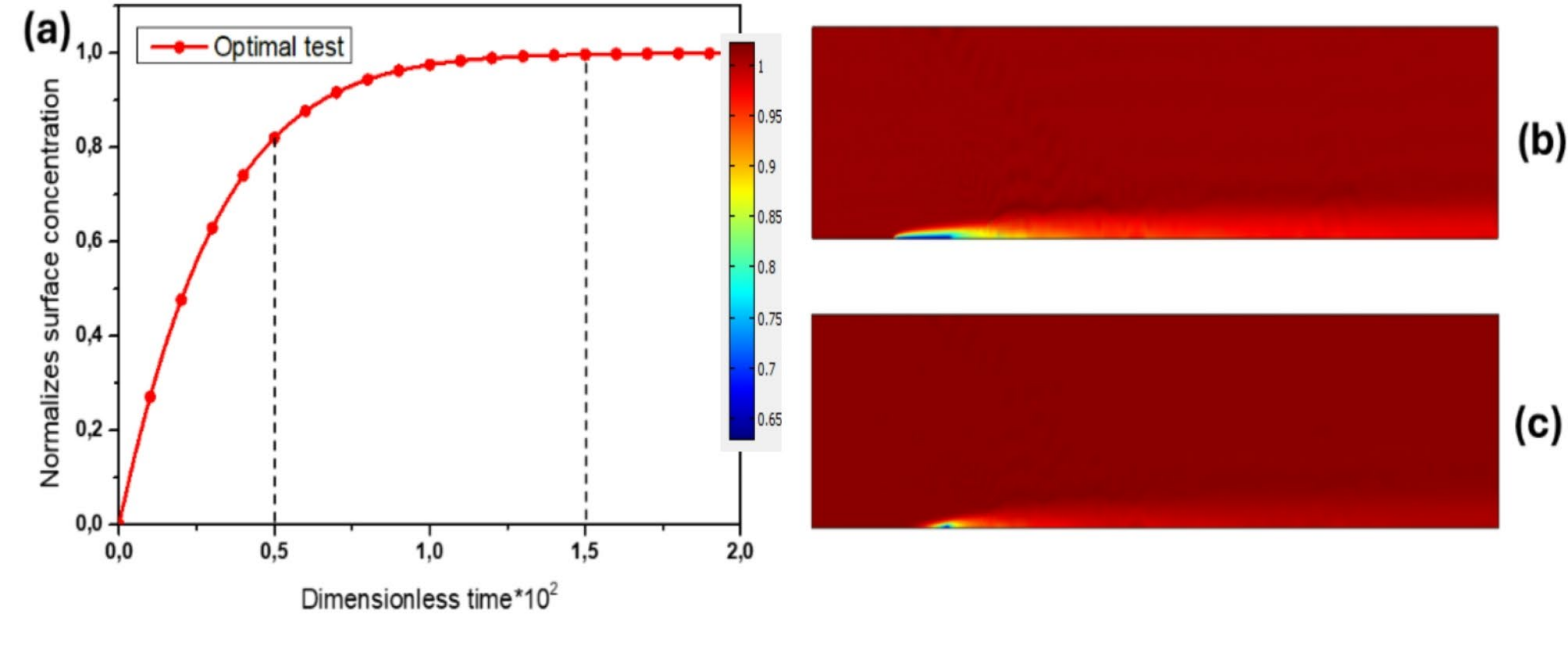
(**a**) Normalized surface concentration for the optimal test. (**b**) and (**c**) diffusion boundary layers during the adsorption phase at two dimensionless times, T_R_= 50 and T_R_=150, respectively.

Figure 12a shows the normalized complex concentration for the optimal test using the optimized parameters, while Fig. 12b,c illustrate the antigen diffusion boundary layers near the reaction surface at dimensionless adsorption times of 50 and 150, respectively. Notably, at t = 150 (where t is the dimensionless saturation time), the diffusion boundary layer thickness is remarkably thin, indicating efficient mass transport for the analyte-ligand bond in the optimal test. This improvement in mass transport efficiency contributes to the enhanced performance of the biosensor.

### ANN-MLP predictions

The Multi-Layer Perceptron Artificial Neural Network (ANN-MLP) is a type of ANN distinguished by its interconnected layers, including input, hidden, and output layers, widely applied in machine learning tasks like classification and pattern recognition^53,54^. Learning in ANN-MLP occurs through weight and bias adjustments during training^55^. An exhaustive investigation of 81 simulation data points derived from the Full L_81_(3^4^) design experiment was utilized to train various networks with different quantities of hidden layer neurons. The training process employed back-propagation with gradient descent, dividing the dataset into training (70%), testing (15%), and validation (15%) subsets.

The following coefficients are calculated to control the performance of the proposed models in this study^42,43^. The first coefficient is the value of the root mean square error.

23 $$RMSE=\sqrt{\frac{1}{N}\sum _{i=1}^{N}{\left({y}_{i}-{\widehat{y}}_{i}\right)}^{2}}$$

where N and are numbers of observations.

We can also use the coefficient of determination ${R}^{2}$ to evaluate the performance of the prediction. This coefficient is given by^32^:

24 $${R}^{2}=1-\frac{\sum _{i=1}^{N}{\left({y}_{i}-{\widehat{y}}_{i}\right)}^{2}}{\sum _{i=1}^{N}{\left({y}_{i}-{\overline{y}}\right)}^{2}}$$

where ${\overline{y}}$ is average value of y.

The numerator in the above equation corresponds to the sum of squares of residuals whereas the denominator is related to the variance of the data. The best prediction is obtained when the coefficient of determination ${R}^{2}$ is close to one.

When the number k of input variables of the model increases, the coefficient of determination ${R}^{2}$ automatically increases. To correct this bias, an adjusted coefficient is proposed. It is defined by^56^:

25 $${R}_{Adj}^{2}=1-\left(1-{R}^{2}\right)\frac{N-1}{N-k-1}$$

**Fig. 13:**
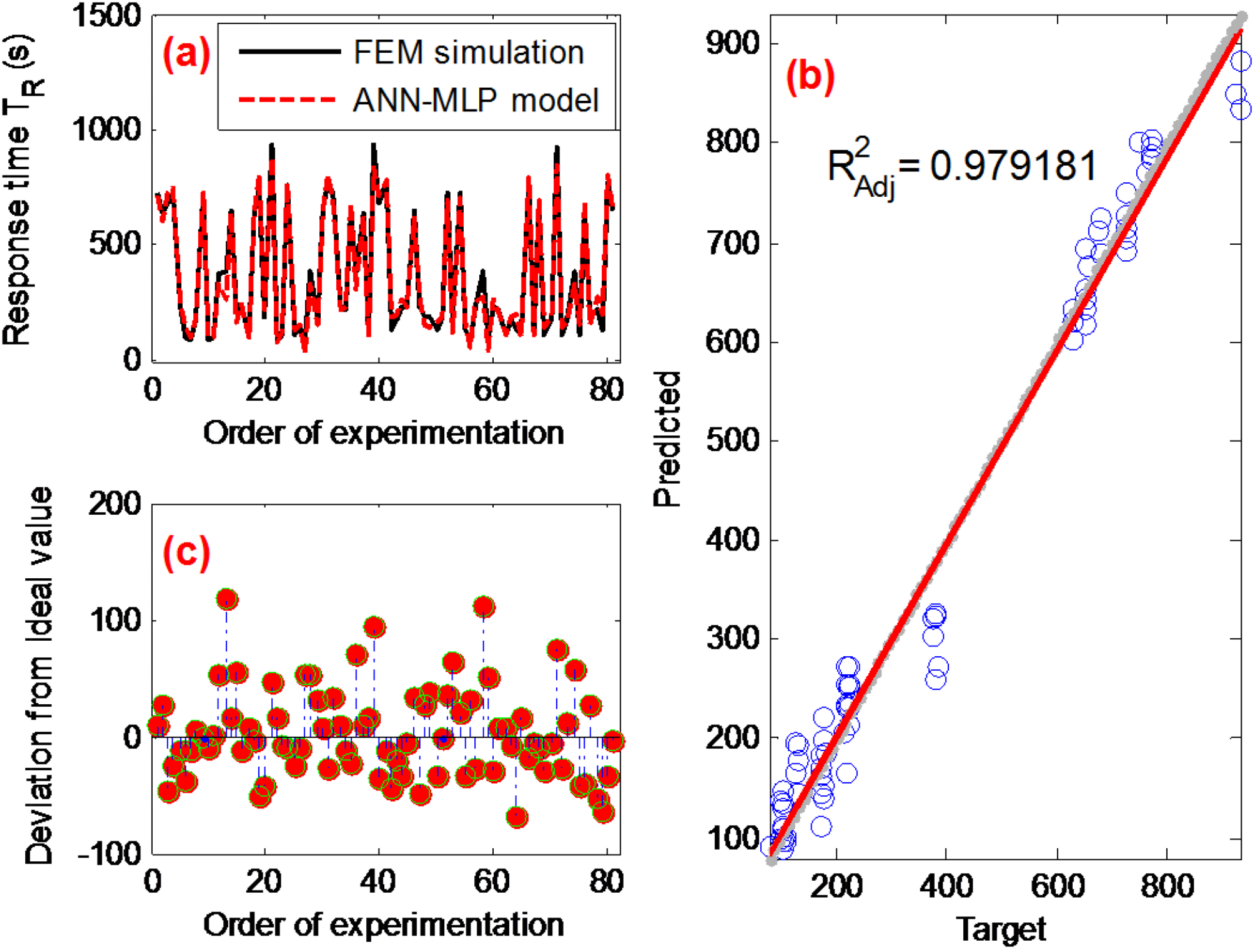
(**a**) Comparison of observed response time values using the ANN-MLP model; (**b**) statistical analysis fit of the ANN-MLP model; (**c**) deviation analysis of the ANN-MLP model predictions from actual response time values.

Figure 13 assesses the ANN-MLP model’s ability to predict the biosensor’s detection time. Subplot (**a**) compares simulated and predicted values, demonstrating the alignment between model predictions and actual data. Subplot (**b**) presents a statistical analysis fit, evaluating the degree of fit between predicted and actual values. In subplot (**c**), the deviation of predicted values from actual values is examined, aiding in understanding predictive errors and discrepancies. With an achieved adjusted coefficient of determination (${R}_{Adj}^{2}$) of 0.97 and a root mean square error (RMSE) of 42.12, the model demonstrates effective predictive capability.

### ANN-PSO predictions

The incorporation of the Particle Swarm Optimization (PSO) algorithm into a traditional Artificial Neural Network (ANN) presents a promising approach. It optimizes the connection weights within the ANN, aiming to identify the optimal values that yield the best results. The PSO algorithm begins by generating a population of particles, each representing a potential solution set to be employed within the neural network^57^. Evaluating the fitness of each particle involves considering local and global information, and this information is retained within each particle. PSO uses this data to update particle velocities and efficiently explore the solution space.

The choice of the ANN-PSO algorithm is based on its ability to efficiently deal with complex and non-linear optimization problems (like our case). The combination of ANN for predictive modeling and PSO for parameter optimization improves the prediction of biosensor performance. This method showed superior results in terms of prediction accuracy and computational efficiency compared to other tested optimization algorithms^31,32^.

**Fig. 14:**
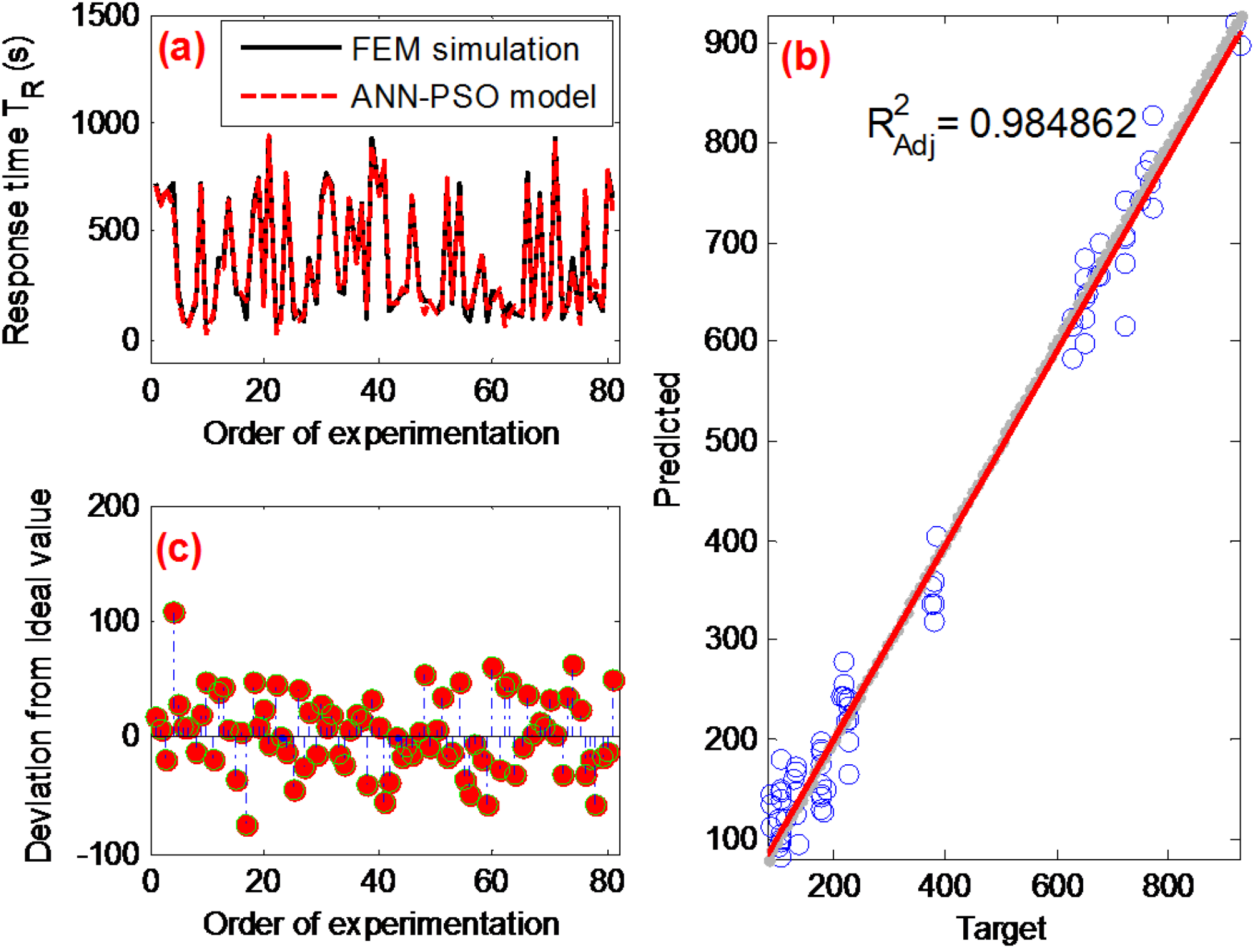
(**a**) Comparison of observed response time values using ANN-PSO model; (**b**) statistical analysis fit of the ANN-PSO model; (**c**) deviation analysis of ANN-PSO model predictions from actual response time values.

Configured with a swarm size of 150, a cognitive coefficient (C_1_) of 1.5, a social coefficient (C_2_) of 2, and an inertia weight (W) of 0.9, the ANN-PSO model delivers highly accurate predictive results, evidenced by an outstanding adjusted regression coefficient of 0.98 (as illustrated in Fig. 14). The performance metrics, including RMSE = 33.2, and R^2^ = 0.99, consistently demonstrate the superiority of the PSO-ANN model in capturing and predicting complex patterns in the Full L_81_(3^4^) given data.

## Conclusion

This study presents a numerical optimization of a microfluidic chip designed for rapid COVID-19 bioassays. By thoroughly analyzing the kinetics of the SARS-CoV-2 binding reaction, we identified four key control parameters: the Reynolds number, Damkohler number, Schmidt number, and the position of the reaction surface. To optimize these key parameters, we used the Taguchi method in conjunction with ANOVA, reducing the number of required simulations from 81 to just 9 using the L_9_(3^4^) orthogonal array. These approaches allowed us to efficiently explore the optimal combination of key parameters and their effects on biosensor performance. The optimal combination corresponds to a Reynolds number (Re) of 0.04 (level 3), a Damkohler number (Da) of 1000 (level 3), a Schmidt number (Sc) of 100,000 (level 3), and a dimensionless reaction surface position (X) of 1 (level 1).

Using the optimized values, the biosensor detection time was significantly reduced to 21 min, demonstrating the potential for rapid COVID-19 detection. Furthermore, the integration of Particle Swarm Optimization (PSO) with an Artificial Neural Network (ANN-PSO) significantly improved the predictive accuracy and robustness of our approach compared to the conventional ANN-MLP model.

This study underscores the effectiveness of combining the Taguchi method, ANOVA, and ANN-PSO for optimizing microfluidic biosensors, paving the way for rapid, efficient, and accurate COVID-19 detection. The proposed methodology not only offers significant improvements in biosensor performance but also holds promise for broader applications in the field of bioassays in general.

## Data Availability

The data that support the findings of this study are available from the corresponding author upon reasonable request.
